# Optimization of amorphadiene production in engineered yeast by response surface methodology

**DOI:** 10.1007/s13205-013-0156-y

**Published:** 2013-07-24

**Authors:** Rama Raju Baadhe, Naveen Kumar Mekala, Sreenivasa Rao Parcha, Y. Prameela Devi

**Affiliations:** 1 Department of Biotechnology, National Institute of Technology, Warangal, 506004 India; 2 Department of Zoology, Kakatiya University, Warangal, 506009 India

**Keywords:** Response surface methodology, *S*. *cerevisiae*, Amorphadiene, Isoprenoids

## Abstract

Isoprenoids are among the most diverse bioactive compounds synthesized by biological systems. The superiority of these compounds has expanded their utility from pharmaceutical to fragrances, including biofuel industries. In the present study, an engineered yeast strain *Saccharomyces cerevisiae* (YCF-AD1) was optimized for production of Amorpha**-**4, 11**-**diene, a precursor of anti-malarial drug using response surface methodology. The effect of four critical parameters such as KH_2_PO_4_, methionine, pH and temperature were evaluated both qualitatively and quantitatively and further optimized for enhanced amorphadiene production by using a central composite design and model validation. The “goodness of fit” of the regression equation and model fit (*R*^2^) of 0.9896 demonstrate this study to be an effective model. Further, this model will be used to validate theoretically and experimentally at the higher level of amorphadiene production with the combination of the optimized values of KH_2_PO_4_ (4.0), methionine (1.49), pH (5.4) and temperature (33 °C).

## Introduction

Isoprenoids (terpenoids) are the most structurally diverse class of natural compounds commonly produced in plants (Croteau et al. 2000). Terpenoids are classified according to their carbon number (basic isoprene (C_5_) unit) as mono (C_10_), sesqui (C_15_), di (C_20_), sester (C_25_), tri (C_30_), tetra (C_40_) and polyterpenoids (C_*n*_) (Ruzicka 1959). More than 55,000 terpenes have been isolated and characterized, consistently doubling in their numbers each decade (Breitmaier 2006; McGarvey and Croteau 1995). Isoprenoids have diverse functional roles in plants such as growth, defense and development (McGarvey and Croteau 1995). Based on these characteristic features, terpenoids have prominence in pharmaceutical, fragrances and biofuel industries (for e.g. bisabolene is an alternative source for jet fuel (Breitmaier 2006; Peralta-Yahya et al. 2012).

Artemisinin is a well-known sesquiterpene lactone peroxide, extracted from the shrub *Artemisia annua*. ‘Artemisininins’ (artemisinin and its derivatives) are recommended by the World Health Organization (WHO) in combination with other effective anti-malarial drugs, known as artemisinin-based combination therapy (ACT) for malarial treatment (Bloland 2001). Since then, the incompetence in large-scale chemical synthesis of artemisinin and enormous demand and price directed the scientific world towards the semi-synthesis of artemisinin followed by microbial production of the precursor amorpha**-**4,11-diene. Heterologous production of amorpha**-**4, 11-diene was first established in *Escherichia coli* by the expression of the mevalonate pathway from yeast and amorpha**-**4, 11-diene synthase (ADS) from *A. annua* (Martin et al. 2003). The production of amorpha-4, 11-diene from *Saccharomyces cerevisiae* revealed that cytochrome P450 enzyme was responsible for the production of artemisinic acid (Mercke et al. 2000; Martin et al. 2003; Ro et al. 2006). Artemisinic acid was produced from yeast by a series of alterations and adjustments to the endogenous mevalonate pathway, such as high-level expression of ADS, overexpression of farnesyl diphosphate synthase (FDPS), expression of the catalytic domain of HMG-CoA reductase(HMGCR), reduced expression of squalene synthase (SQS) and increased expression of *UPC2* allele transcription factor (Ro et al. 2006). Artemisinic acid was produced by a three-step oxidation of amorphadiene, by cytochrome P450 reductase (*A. annua*) (Ro et al. 2006). However, cytochrome P450 reductase instability and lower yields of artemisinic acid compared to amorphadiene drew attention towards improving the production of amorphadiene, the precursor of artemisinic acid in *S. cerevisiae*. (Westfall et al. 2012). In combination with traditional metabolic engineering, we also applied enzyme fusion technology for improved production of amorphadiene in *S. cerevisiae* (YCF-AD-1) (unpublished data). Our previous observations show that in engineered yeast, the mevalonate pathway is tightly regulated by methionine and phosphate levels along with other physical parameters such as pH and temperature. Optimization of these parameters by classical experimental optimization is difficult because it involves changing one variable at a time while keeping the others constant. In addition, it is not practical to carry out experiments with every possible factorial combination of the test variables, because of the large number of experiments required to be done and/or evaluated (Akhnazarova and Kafarov 1982; Myers and Montgomery 1995) which does not emphasize the effect of interactions among various parameter. Besides this, it will be a tedious and time-consuming process, especially when there are a large number of parameters to take into consideration. An alternative and more efficient approach is the use of the statistical method to resolve this kind of practical hurdles. Response surface methodology (RSM) has been widely used to evaluate and understand the interactions between different process parameters (Khuri et al. 1987). RSM was applied successfully for optimizing process parameters for various processes in biotechnology, from biological treatment of toxic wastes (Ravichandra et al. 2008a, b) to enzyme production (Doddapaneni et al. 2007; Tatineni et al. 2007; Ravichandra et al. 2008a, b; Chennupati et al. 2009) including recombinant products (Vellanki et al. 2009; Farhat-Khemakhem et al. 2012). Till date, studies with statistical optimization of parameters for production of amorphadiene have not been reported elsewhere. Our present work emphasizes the key parameters (KH_2_PO_4_, methionine, pH and temperature) affecting amorpha-4,11-diene production in engineered *S. cerevisiae* strain (YCF-AD-1), optimized using RSM.

## Materials and methods

### Microbial strain and inoculum preparation

The yeast strain *S. cerevisiae* (YCF-AD-1) used in this study was developed in our previous studies (unpublished data) and originated from *S. cerevisiae* MTCC 3157. The strain was cultured in 250 mL Erlenmeyer flasks containing 100 mL medium with the following composition (g/L): galactose, 20; (NH_4_)_2_.SO_4_, 7.5; MgSO_4_.7H_2_O, 0.5; trace metals solution, 2 mL; vitamins solution, 1 mL and 50 μl/L silicone anti**-**foam. The pH of the media was adjusted to 5.0 using 1 M NaOH and further autoclaved. Filter-sterilized vitamin solution and galactose solution were aseptically added to the sterile medium. The flasks were incubated for 24 h at 28 ± 2 °C at 150 rpm.

### Amorphadiene production

The media components KH_2_PO_4_ and methionine were added according to experimental designs (Table 2) to the minimal medium (Verduyn et al. 1992) which consisted of (g/L): galactose, 20; (NH_4_)_2_SO_4_, 5; MgSO_4_.7H_2_O, 0.5; EDTA, 0.015; ZnSO_4_.7H_2_O, 0.0045; CoC1_2_.6H_2_O, 0.0003; MnC1_2_. 4H_2_O, 0.001; CuSO_4_.5H_2_O, 0.0003; CaC1_2_.2H_2_O, 0.0000045; FeSO_4_.7H_2_O, 0.0003; NaMoO_4_.2H_2_O, 0.0004; H_3_BO_3_, 0.001; KI, 0.0001; 25 μl/L silicone anti**-**foam (Merck). It was autoclaved and cooled to room temperature. The filter solution was added to this sterile medium (Dynesen et al. 1998). The pH was adjusted according to the experimental design (Table 2). Aseptically, 1 % of inoculum was added to the flask, mixed thoroughly and incubated at the temperature specified in the experimental designs (Table 1) for 80 h at 150 rpm. After cells reached OD600 value of 1.0, 20 % (v/v) of isopropyl myristate (Merck Millipore, Germany) was added aseptically to the media. This isopropyl myristate layer was sampled and diluted with ethyl acetate for determination of amorphadiene by gas chromatography coupled with mass spectrometry GC–MS (Agilent Technologies, USA).

**Table 1:** Range and levels of the variables in coded units for response surface methodology studies

Variables	−2	−1	0	+1	+2	ΔX
KH_2_PO_4_ (*x*_1_)	0	4	8	12	14	4
Methionine (*x*_2_)	0	1	2	3	4	1
pH, 5.5 (*x*_3_)	4.0	4.5	5.0	5.5	6.5	0.5
Temperature, °C (*x*_4_)	25	27	32	37	39	2

## Analytical methods

### Amorpha**-**4, 11-diene analysis

Amorpha**-**4, 11-diene was analysed by gas chromatography with flame-ionization detection (GC–FID). Samples from flasks were centrifuged at 5,000 rpm for 5 min and diluted directly into ethyl acetate and mixed for 30 min on a vortex mixer. After phase separation, 0.6 mL of the ethyl acetate layer was transferred to a capped vial for analysis. The ethyl acetate-extracted samples were analysed using the GC–FID with a split ratio of 1:20 and separated using a DB-WAX column (50 m × 200 μm × 0.2 μm) with hydrogen as carrier gas with a flow rate of 1.57 mL/min. The temperature program for the analysis was as follows: the column was initially held at 150 °C for 3 min, followed by a temperature gradient of 5 °C per min to a temperature of 250 °C. Amorpha**-** 4, 11-diene peak areas were converted to concentration values from external standard calibrations using *trans***-**caryophyllene standard (Westfall et al. 2012).

### Experimental design and response optimization

Response optimization method was used to increase the yield of amorphadiene by using RSM. On the basis of previous experience (unpublished data), four critical parameters for amorphadiene production were selected and further evaluated for their interactive behaviour by using statistical approach. The levels of the four medium variables, KH_2_PO_4_, 6.5(*x*_1_); methionine, 1.5(*x*_2_); pH, 5.5(*x*_3_); and temperature, 32 °C (*x*_4_), were selected as central points, and each variable was coded at five levels, −2, −1, 0, +1 and +2, using Eq. (1). For statistical calculations, the centre variable *X*_*i*_ was coded as *x*_*i*_ according to the following transformation. The range and levels of the variables in coded units for RSM studies are given in Table 1.

1 $$x_{i}=X_{i}-X_{0}/\Delta X$$

where *x*_*i*_ is the dimensionless coded value of the variable *X*_*i*_, *X*_0_ represents the value of *X*_*i*_ at the centre point and Δ*X* the step change. The behaviour of the system is explained by the following quadratic model [Eq. (2)].

2 $$Y=\beta_{0}+\sum \beta_{i}X_{i}+\sum \beta_{\mathrm{ii}}X_{i}^{2}+\sum \beta_{\mathrm{ij}}X_{i}X_{j}$$

where *Y* is the predicted response, *β*_0_ is the intercept term, *β*_*i*_ the linear effect, *β*_*ii*_ the squared effect and *β*_*ij*_ the interaction effect. The full quadratic equation for four factors is given by the following model [Eq. (3)].

3 $$\begin{array}{c} Y=\beta_{0}+\beta_{1}X_{1}+\beta_{2}X_{2}+\beta_{3}X_{3}+\beta_{4}X_{4}+\beta_{11}X_{1}^{2}+\beta_{22}X_{2}^{2}+\beta_{33}X_{3}^{2}+\beta_{44}X_{4}^{2}+\beta_{12}X_{1}X_{2}\\ \;\;\;\;\;\;+\beta_{13}X_{1}X_{3}+\beta_{14}X_{1}X_{4}+\beta_{23}X_{2}X_{3}+\beta_{24}X_{2}X_{4}+\beta_{34}X_{3}X_{4} \end{array}$$

Previous experimental studies have considered such models using central composite design (CCD) (Cochran and CoxIn 1957; Montgomery 2001). In this study, a 2^4^ full-factorial design with eight star points and six replicates at the central points were employed to fit the second-order polynomial model, where we carried out a set of 30 experiments. Data obtained in the above experiments were analysed for regression, and graphical analysis using Design Expert^®^ software (Stat-Ease Inc, USA) was used for regression and graphical analysis of the data obtained. The optimal combination of variables for the amorphadiene production were analysed using CCD experiments and were tabulated in Table 2. Table 2 shows the results of CCD experiments used for studying the effect of four independent variables along with the mean predicted and experimental responses. Each response was analysed, and a second-order regression model was developed. The model was validated in each case, and a set of optimal values were calculated.

**Table 2:** Design of experiments by central composite design for response surface methodology studies

Std. order	Run order	*x*_1_	*x*_2_	*x*_3_	*x*_4_	Coefficients assessed by	Amorphadiene (mg/L) Experimental	Amorphadiene (mg/L) Predicted
1	14	−1	−1	−1	−1	Full-factorial 2^4^ design (16 expts)	41.98	44.31
2	10	1	−1	−1	−1		40.12	38.01
3	22	−1	1	−1	−1		46.24	46.22
4	8	1	1	−1	−1		42.37	39.63
5	30	−1	−1	1	−1		48.24	41.71
6	2	1	−1	1	−1		39.21	41.31
7	29	−1	1	1	−1		46.21	42.04
8	9	1	1	1	−1		40.35	41.35
9	26	−1	−1	−1	1		68.24	63.09
10	1	1	−1	−1	1		48.25	52.31
11	18	−1	1	−1	1		58.23	56.02
12	3	1	1	−1	1		42.58	44.96
13	21	−1	−1	1	1		58.24	60.87
14	11	1	−1	1	1		60.12	55.99
15	15	−1	1	1	1		54.27	52.23
16	25	1	1	1	1		49.5	47.06
17	4	−2	0	0	0	Star points (8 expts)	175	190.15
18	17	2	0	0	0		182.54	184.42
19	27	0	−2	0	0		74.21	81.00
20	20	0	2	0	0		67.25	77.49
21	19	0	0	−2	0		174.35	177.81
22	16	0	0	2	0		164	177.57
23	24	0	0	0	−2		159.77	169.90
24	28	0	0	0	2		175.24	182.14
25	7	0	0	0	0	Central points (6 expts)	205.34	190.77
26	23	0	0	0	0		201.27	190.77
27	12	0	0	0	0		198.24	190.77
28	6	0	0	0	0		195.28	190.77
29	13	0	0	0	0		197.32	190.77
30	5	0	0	0	0		198.25	190.77

## Results and discussion

### Multiple responses optimization and building model

RSM is a sequential and effective procedure where the primary objective of the methodology is to run rapidly and efficiently along the path of enhancement towards the general vicinity of the optimum, identifying the optimal region for running the process (Mekala et al. 2008; Chennupati et al. 2009; Potumarthi et al. 2012). The four independent variables such as KH_2_PO_4_, methionine, pH and temperature were chosen for optimized production of amorphadiene and experiments were performed according to the given CCD experimental design (Table 2), to obtain optimal combination of variables for the process. Thirty experimental runs with different combinations of four factors were carried out. For each run, the experimental responses along with the predicted response were calculated from the regression Eq. (4).

4 $$\begin{array}{c} Y=190.777-2.867X_{1}-1.756X_{2}-0.123X_{3}+6.121X_{4}-0.0719X_{1}X_{2}+1.4744X_{1}X_{3}\\ \;\;\;\;\;\;-1.1194X_{1}X_{4}-0.3944X_{2}X_{3}-2.243X_{2}X_{4}+0.0956X_{3}X_{4}-3.481X_{1}^{2}-111.521X_{2}^{2}\\ \;\;\;\;\;\;-13.075X_{3}^{2}-14.7455X_{4}^{2} \end{array}$$

where, *Y* is the predicted response, and *x*_1_, *x*_2_, *x*_3_ and *x*_4_ are coded values of KH_2_PO_4_, methionine, pH and temperature, respectively. The regression equation was used to calculate the predicted responses given in Table 2, and assessment of the predicted values with the experimental values indicated that these data were in reasonable agreement. The maximum response (205.34 mg/L) was obtained in run number 7, and in general all the runs with middle levels of parameters gave higher yields compared to other combinations. The data were analysed by regression analysis, and the optimized values to maximize the responses were observed at 4, 1.49, 5.47 and 33.13 for KH_2_PO_4_, methionine, pH and temperature, respectively.

Suitability of the model was confirmed by the analysis of variance (ANOVA) using Design Expert software and the results are shown in Table 3. ANOVA of the quadratic regression model suggests that the model is significant with a computed *F* value of 101.6917 and a *P* > *F* value less than 0.05. A lower value for the coefficient of variation suggests higher consistency of the experiment, and in this case the obtained CV value of 9.19 % demonstrates a greater reliability of the trials. *R*^2^ is the coefficient of variance of response under test and whose values are always between 0 and 1; closer the value of *R*^2^ to 1, the stronger is the statistical model and better is the prediction of response (Myers and Montgomery 1995). The coefficient of determination (*R*^2^) for response of amorphadiene is 0.9896 (Table 3), indicating that the statistical model can explain 98.96 % of variability in the response and only 1.04 % of the variations for amorphadiene not explained by the model. The adjusted *R*^2^ value corrects the *R*^2^ value for the sample size and for the number of terms in the model. The value of the adjusted determination coefficient (Adj *R*^2^) for amorphadiene (0.9798) is also good, supporting the significance of this developed model (Cochran and CoxIn 1957). The significance of individual variables can be evaluated from their *P* values, with the more significant terms having a lower *P* value (Table 4). The values of *P* > *F* less than 0.05 indicate that the model terms are significant and in this case *X*_4_, *X*_2_^2^, *X*_3_^2^ and *X*_4_^2^ were found to be significant model terms and there were no significant interactions between the parameters.

**Table 3:** Model summary and analysis of variance for the quadratic model

Source of variations	Sum of squares	Degree of freedom	Mean square	*F* value	Probability (*P*)
Regression	132,761.320	14	9,482.95	101.69	<0.0001
Residual	1,398.780	15	93.25		
Total	134,160.099	29			

*R* = 0.9947, *R*^2^ = 0.9896, adjusted *R*^2^ = 0.9798, CV = 9.19 %

**Table 4:** Model coefficients estimated by multiple linear regressions (significance of regression coefficients)

Model term	Coefficient estimates	Standard error	*F* value	*P* value Prob > *F*
Intercept	190.767	2.99967	101.692	<0.0001
*x*_1_	−2.8672	2.27611	1.58686	0.227
*x*_2_	−1.7561	2.27611	0.59528	0.4524
*x*_3_	−0.1233	2.27611	0.00294	0.9575
*x*_4_	6.12111	2.27611	7.23228	0.0168^a^
*x*_1_ *x*_2_	−0.0719	2.41418	0.00089	0.9766
*x*_1_ *x*_3_	1.47438	2.41418	0.37297	0.5505
*x*_1_ *x*_4_	−1.1194	2.41418	0.21499	0.6495
*x*_2_ *x*_3_	−0.3944	2.41418	0.02669	0.8724
*x*_2_ *x*_4_	−2.2431	2.41418	0.86332	0.3675
*x*_3_ *x*_4_	0.09563	2.41418	0.00157	0.9689
*x*_1_^2^	−3.4805	5.99933	0.33658	0.5704
*x*_2_^2^	−111.52	5.99933	345.545	<0.0001^a^
*x*_3_^2^	−13.076	5.99933	4.75021	0.0456^a^
*x*_4_^2^	−14.746	5.99933	6.04108	0.0266^a^

^a^Significant at *P* < 0.05

**Fig. 1:**
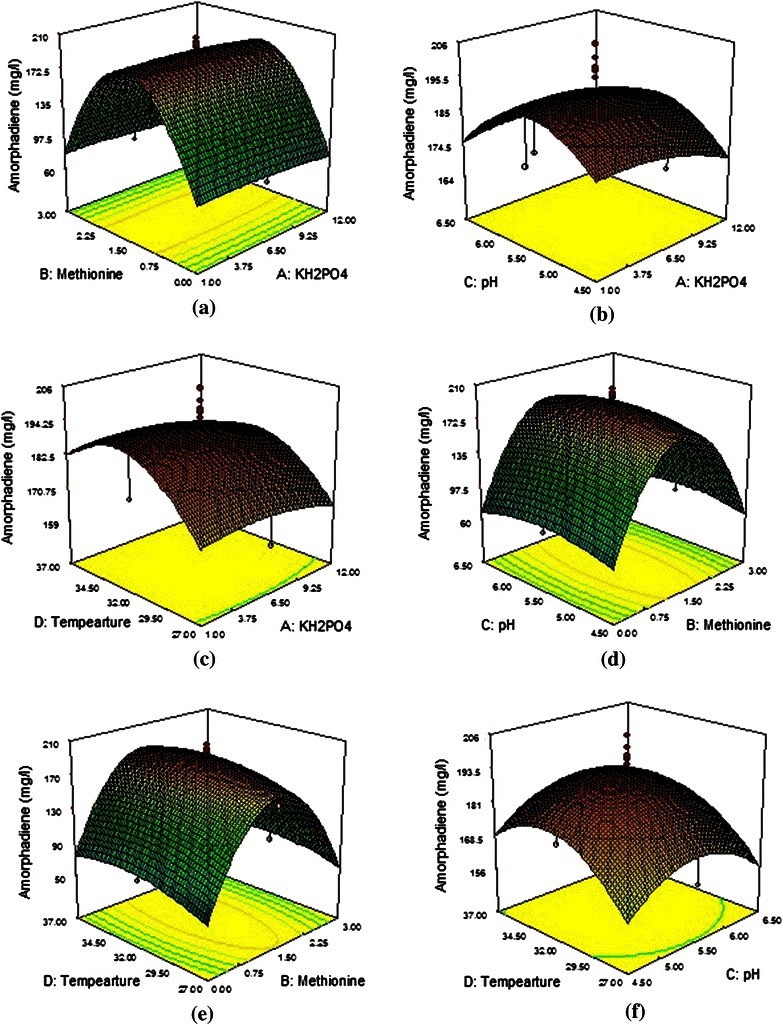
**a**–**f** 3-D surface and contour plot of amorphadiene production by *S. cerevisiae* (mg/L): the effect of two variables while the other two were held at 0 level

Surface plots are generally the graphical representation of the regression equation for identifying the optimal levels of each parameter for attaining the maximum response (amorphadiene) production. Figure 1a–f shows the response surfaces obtained for the interaction effects of tested variables. In each response graph, the effect of the two variables on amorphadiene production was shown when the other two variables were kept constant. Figure 1a shows the interaction relationship between the two independent variables, namely, KH_2_PO_4_/methionine and their effects on amorphadiene production .

It was observed from Fig. 1a that amorphadiene synthesis was significantly affected by methionine concentration. Amorphadiene synthesis was increased with increase in methionine concentration up to 1.5 mM and further increase in methionine concentration did not show any influence on amorphadiene production, whereas the addition further resulted in decreased production. The same pattern was observed in other graphs (Fig. 1d, e). This indicates that the increase in the methionine concentration tightly regulates the engineered repressible methionine promoter in *S. cerevisiae* by limiting the conversion of farnesyl pyrophosphate into squalene (Asadollahi et al. 2008).

Studies on the effect of varied methionine concentration (0–2 mM) with engineered yeast reported approximately 125 mg/L of amorphadiene with 0.2 mM methionine concentration. In previous studies, 1.5 and 2 mM concentrations of methionine were considered for the production of plant sesquiterpenes in yeast during batch and fed-batch operations, respectively (Asadollahi et al. 2008; Paradise et al. 2008). But these reported studies were not statistically optimized for methionine concentration; in the present work, it was observed that 1.49 mM of methionine was the optimum concentration with combinations of other optimum variables leading to synthesis of 191.5 mg/L of amorphadiene. The effect of KH_2_PO_4_ did not have significant effect in combination with methionine concentration, but there was significant effect observed in combination with the other two variables, temperature and pH (Fig. 1a, b and c). There was a significant increase in amorphadiene production with increase in KH_2_PO_4_ concentration up to 6.5 g/L and further increase in its concentration did not show any significant improvement in amorphadiene production. Previous studies reported that low phosphate concentration improved amorphadiene production, which may be by limiting the growth and channelling the carbon flux towards amorphadiene production (Westfall et al. 2012). In this study, 4.01 g/L of KH_2_PO_4_ was the recommended concentration for the optimized production of amorphadiene in combination with other optimized parameters.

Figure 1b, d, f shows the effect of pH on amorphadiene production in combination with KH_2_PO_4_ and temperature. There is increase in amorphadiene production with increase in pH and the maximum production was at pH 5.5. In previous studies, the production of plant sesquiterpenes in yeast was carried out at pH 6.50, 5 ± 0.5, 5.0 for shake flasks, batch and fed-batch cultivation, respectively (Asadollahi et al. 2008), whereas the enzyme responsible for amorphadiene production (amorphadiene synthase) showed optimum activity at varied pH 6.5–7.5 levels in *artemisia annua* (Bouwmeester et al. 1999; Mercke et al. 2000; Picaud et al. 2005; Picaud et al. 2007). In this study, *S. cerevisiae* showed optimum pH as 5.5 and the present model gave 5.47 as an optimum value along with other optimal parameters.

The effects of temperature in response to combination with other variables, KH_2_PO_4_, methionine and pH, are shown in Fig. 1c, e, f. At low temperature (27 °C), amorphadiene synthesis was very less and increased with increment in temperature up to 33 °C. There was a rapid increase in amorphadiene production in combination with KH_2_PO_4_ and pH, whereas in combination with methionine the effect of temperature was not significant. Based on this model, the optimal combination of all parameters is KH_2_PO_4_, 4.01; methionine, 1.49; pH, 5.47; temperature 33.13 °C with a predicted response value of 192.119 mg/L. Experiments conducted with the same optimal conditions, such as KH_2_PO_4_, 4.0; methionine, 1.49; pH, 5.4; temperature 33 °C, yielded 191.5 mg/L of amorphadiene, which resembles closely the predicted response. Finally, these results suggest that methionine has a high significant effect on amorphadiene production compared to other variables. Hence, the maximum amorphadiene production can be achieved with a relatively limited number of experimental runs using the appropriate statistical design and optimization technique.

## Conclusion

The use of RSM with a full-factorial rotatable CCD for determination of optimal medium and physical parameters for amorphadiene production was demonstrated using the essential parameters. The use of this methodology will be successful for any combinational analysis, in which an analysis of the effects and interactions of many experimental factors are required. Rotatable central composite experimental design maximizes the amount of information that can be obtained while limiting the number of individual experiments. Thus, smaller and less time**-**consuming experimental designs could generally be sufficient for optimization of many such fermentation processes (Tatineni et al. 2007). The superiority of terpenoids has expanded their utility from pharmaceutical to fragrances, including biofuel industries. Significant efforts have been made for establishing microbial cell factories for the production of a wide variety of high value-added chemicals. However, there are some difficulties for the large-scale production of these chemicals. In addition to the synthetic biology and metabolic engineering approaches, statistical optimization methods will provide insights into the production of high value-added chemicals. In the present study, the overall view on the optimization of the process using essential parameters for amorphadiene production provides insights into the process development and further scaling-up process. The results of ANOVA and regression of the second-order model showed that the linear effects of temperature and the interactive effects of the three variables, methionine, pH and temperature, were significant for amorphadiene production. Among these three variables, methionine has a more significant interactive effect. Finally, we conclude our study by stating that the optimization of amorphadiene production was by the second-order model, and ANOVA requires optimal conditions of: KH_2_PO_4_, 4.0; methionine, 1.49; pH, 5.4; temperature 33 °C.
